# A PI3K Inhibitor with Low Cardiotoxicity and Its Synergistic Inhibitory Effect with Gilteritinib in Acute Myelogenous Leukemia (AML) Cells

**DOI:** 10.3390/molecules30112347

**Published:** 2025-05-27

**Authors:** Tianze Wu, Yi Chen, Yimin Gong, Mingzhu Lu, Chengbin Yang, Yannan Yang, Yun Ling, Yaming Zhou

**Affiliations:** 1 Department of Chemistry, Fudan University, Shanghai 200433, Chinayunling@fudan.edu.cn (Y.L.); 2 South Australian immunoGENomics Cancer Institute, The University of Adelaide, Adelaide, SA 5005, Australia

**Keywords:** 7-azaindazole, PI3K inhibitor, cardiotoxicity, oxidative stress, acute myelogenous leukemia, combination therapy

## Abstract

*N*-(2-chloro-5-(3-(pyridin-4-yl)-1H-pyrazolo [3,4-*b*]pyridin-5-yl)pyridin-3-yl)-4-fluorobenzenesulfonamide, namely, FD274, is a promising 7-azaindazole-based PI3K inhibitor candidate with high antitumor efficacy against acute myeloid leukemia and reduced cardiotoxicity in the zebrafish model. To advance its clinical translation, in this work, we conducted comprehensive assessments of the cardiotoxicity of FD274 and preliminarily investigated its synergistic antitumor effects with an FLT3 inhibitor, Gilteritinib. The cardiotoxicity profile of FD274, as well as its bioisostere FD268 (positive control), was evaluated using the C57BL/6 mouse model and the H9C2 cell line. The cardiotoxicity of FD274 after a consecutive 20-day treatment period was further assessed in an HL-60 xenograft mouse model. The synergistic cytotoxicity of FD274 with Gilteritinib was evaluated in the HL-60 cell line and the FLT3-ITD cell line MV-4-11. FD274 demonstrated lower adverse effects associated with cardiac dysfunction, oxidative stress, and myocardial injury in the C57BL/6 mouse model and in the H9C2 cell line as compared with FD268. Its negligible adverse effect was further validated in the HL-60 xenograft mice after the 20-day treatment process. Moreover, FD274 demonstrated a synergistic pro-apoptotic effect with Gilteritinib in both HL-60 and MV-4-11 cells. Our findings confirmed the low cardiotoxicity of FD274 and its great potential for combination therapy with Gilteritinib, warranting further development.

## 1. Introduction

Drug-induced cardiotoxicity takes the blame for up to 33% of drug failures, which is one of the major reasons for the withdrawal of drugs from the market [1,2]. The PI3K pathway serves as a therapeutic target for various types of cancer, including chronic lymphocytic leukemia (CLL), breast cancer, and acute myelogenous leukemia (AML), but, at the same time, plays a cardioprotective role, which raises concerns about the cardiotoxic risks of PI3K inhibitors [3,4]. In fact, the clinical use of several PI3K inhibitors such as copanlisib, alpelisib, BKM120, and so forth was hampered primarily due to their cardiac-related toxicities [3,5,6]. In this context, a comprehensive assessment of the cardiotoxicity of promising PI3K inhibitors in the early development stage is of great importance. One of the main mechanisms of cardiotoxicity caused by antitumor drugs is the induction of oxidative stress [7,8,9]. Rather than a simple imbalance between the production and clearance of reactive oxygen species (ROS), oxidative stress is related to the dysfunction of enzymes such as NADPH oxidases that are involved in ROS production [10]. The PI3K/Akt pathway participates in the regulation of the oxidative stress response [11,12,13], promoting the investigation of whether its inhibition would heighten the susceptibility of cardiomyocytes to oxidative stress and subsequently result in cardiotoxicity.

Along with the structural modification strategies used in drug design and development, combination therapy is another effective strategy to enhance efficacy and reduce the risk of adverse effects. Additionally, multi-targeted drug combinations can overcome drug resistance developed during cancer therapy [14,15]. Fms-like tyrosine kinase 3 (FLT3), one of the receptor tyrosine kinases (RTKs), is another therapeutic target for AML. The internal tandem duplication mutation (FLT3-ITD mutation) is one of the most common mutation types of FLT3, which is detectable in approximately 23% of AML cases [16]. The ITD mutation is characterized as inducing the constitutive activation of the tyrosine kinase function, which transduces signals promoting cell growth and survival signals such as AKT, STAT5, and ERK [16,17]. Gilteritinib (ASP2215, Xospata), a dual inhibitor of FLT3/AXL, was approved by the FDA for relapse/refractory (R/R) AML [18,19]. However, the transient and partial responses due to the acquired drug resistance highlight the importance of the development of combinatorial therapeutic approaches [20]. It has been reported that the inhibition of the PI3K/AKT/mTOR pathway displayed synergistic antitumor effects with FLT3 inhibitors [17], which encouraged us to test the therapeutic efficacy of the combination of Gilteritinib and PI3K/AKT/mTOR inhibitors.

Compound *N*-(2-chloro-5-(3-(pyridin-4-yl)-1H-pyrazolo [3,4-*b*]pyridin-5-yl)pyridin-3-yl)-4-fluorobenzenesulfonamide (FD274) is representative of 7-azaindazole-based PI3K inhibitors, and it was selected for its high potency against PI3Ks, with high antitumor efficacy against acute myeloid leukemia [21,22]. Additionally, from the viewpoint of the structure–cardiotoxicity relationship regarding informing the design strategy of novel PI3K inhibitors, we preliminarily proved the low, structure-dependent cardiotoxicity of FD274 using zebrafish embryos [23]. The present study further aimed to perform a comprehensive assessment of the cardiotoxicity of FD274 for its potential clinical translation and to preliminarily investigate its synergistic antitumor effects with Gilteritinib in the HL-60 cell line and the FLT3-ITD cell line MV-4-11.

## 2. Results

### 2.1. The Cardiotoxicity of FD274 and FD268 in C57BL/6 Mouse Model

**Figure 1:**
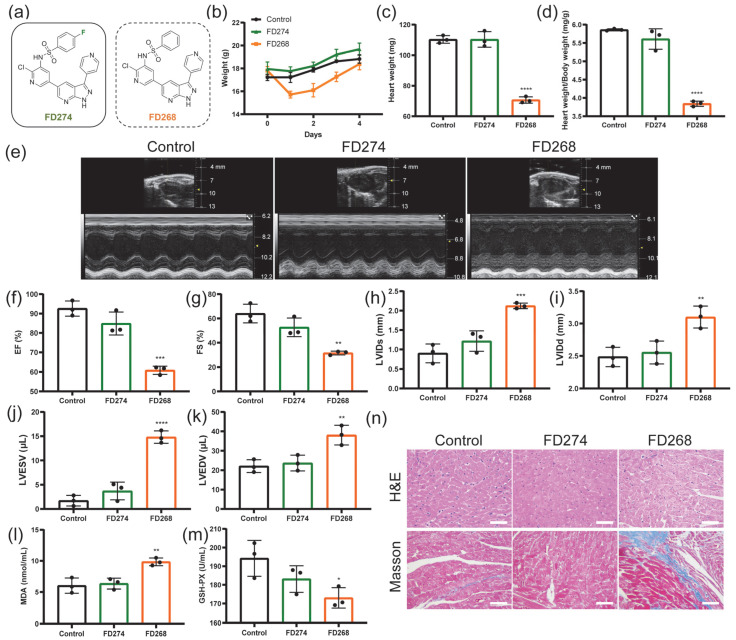
The cardiotoxicity of FD274 and FD268 in C57BL/6 mice (n = 3). (**a**): The structure of FD274, as well as the positive control FD268. (**b**): The body weight change in the mice after the treatment with vehicle (control), FD274, or FD268. (**c**,**d**): The heart weight (**c**) and heart/body weight ratio (**d**) of mice on the fourth day after being treated with vehicle (control), FD274, and FD268. (**e**): Representative M-mode echocardiographic images of the mice acquired on the fourth day after being treated with vehicle (control), FD274, and FD268. (**f**–**k**): Cardiac function parameters of mice in each group. (**l**,**m**): Levels of MDA (**l**) and GSH-PX (**m**) in the mice on the fourth day after being treated with vehicle (control), FD274, and FD268. (**n**): H&E and Masson’s trichrome staining of the heart sections of mice after being treated with vehicle (control), FD274, and FD268. EF: ejection fraction; FS: fractional shortening; LVID: left ventricular internal diameter; LVESV: left ventricular end-systolic volume; LVEDV: left ventricular end-diastolic volume; MDA: malondialdehyde; GSH-PX: glutathione peroxidase. Scale bar = 50 μm. * *p* < 0.05, ** *p* < 0.01, *** *p* < 0.001, **** *p* < 0.0001.

In our previous work, the pharmacokinetic profile of FD274 was investigated at an intravenous dose of 2 mg/kg and an oral dose of 10 mg/kg in SD male rats [22] (Table S1). On this basis, whether FD274 would induce cardiac dysfunction was investigated in a C57BL/6 mouse model. FD268, a bioisostere of FD274 that presented unacceptable cardiotoxicity, was chosen as the positive control to provide more information on the cardiotoxicity of 7-azaindazole-based PI3K inhibitors. As shown in Figure 1b, the body weight of the FD274 group and the control group increased steadily, while that of the FD268 group underwent a sharp decrease followed by a slow recovery. No significant difference in heart weight or heart/body weight ratio was observed between the control group and the FD274 group, while the FD268 group showed a significant decrease in heart weight and heart/body weight ratio compared with the control group (*p* < 0.0001) (Figure 1c,d). The decline in cardiac function, visualized by M-mode echocardiography, is one of the major standards for reporting cardiotoxicity induced by antitumor drugs in mouse models [24,25]. As shown in Figure 1e–k, the FD274 group showed no significant changes in left ventricular internal dimension systole (LVIDs), left ventricular internal dimension diastole (LVIDd), left ventricular end-systolic volume (LVESV), left ventricular end-diastolic volume (LVEDV), ejection fraction (EF), or fractional shortening (FS) compared with the control group, indicating preserved cardiac function. By contrast, the FD268 group showed a significant increase in LVIDs, LVIDd, LVESV, and LVEDV and a significant decrease in EF and FS, indicating cardiac dysfunction. Additionally, neither FD274 nor FD268 affected the left ventricular wall dimension (Figure S1). These results confirmed the lower toxic effects of FD274 in inducing cardiac dysfunction compared with FD268.

The malondialdehyde (MDA) and the glutathione peroxidase (GSH-PX) levels in the mouse serum were examined as indicators of oxidative stress. Mice were subjected to i.p. administration of FD274 and FD268. As shown in Figure 1l,m, FD274 did not increase the level of MDA or GSH-PX. By contrast, FD268 caused a significant increase in the MDA level (*p* < 0.01), which suggests a high lipid peroxidation level. Meanwhile, FD268 caused a significant decrease in the activity of glutathione oxidase (*p* < 0.05), which suggests an impaired antioxidant capacity. H&E and Masson’s trichrome staining of cardiac tissue sections was then performed to visualize myocardial histological alterations. As shown in Figure 1n, the FD274 group exhibited preserved cardiomyocyte morphology, while the FD268 group exhibited extensive cytoplasmic vacuolization as well as the deposition of blue collagen, which suggests myocardial injury induced by FD268. Additionally, neither FD274 nor FD268 induced myocardial apoptosis (Figure S2). The toxic effects of FD274 and FD268 on other organs were also investigated. As shown in Figure S3, the renal tissue section in the FD268 group exhibited glomerulus atrophy, tubular vacuolization, and epithelial cell detachment. The hepatic tissue section in the FD268 group exhibited inflammatory infiltration in the central veins of hepatic lobules. No obvious toxic effect was observed in the organs in the FD274 group. All these results confirmed the reduced cardiotoxicity of FD274.

### 2.2. The Cytotoxic Effects of FD274 and FD268 in H9C2 Cell Line

**Figure 2:**
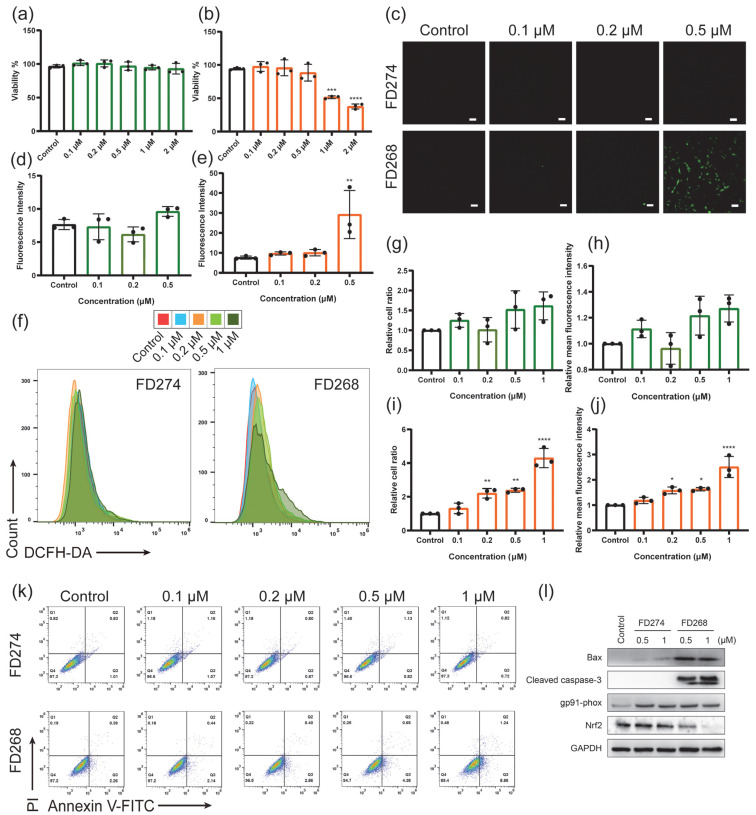
The cytotoxic effects of FD274 and FD268 in the H9C2 cell line. (**a**,**b**): Cell viability of H9C2 cells after being treated with various concentrations of FD274 (**a**) and FD268 (**b**) (n = 3). (**c**): Representative CLSM images of H9C2 cells stained by DCFH-DA after being treated with various concentrations of FD274 and FD268, scale bar = 100 μm. (**d**,**e**): The fluorescence intensity of CLSM images of the FD274 group (**d**) and the FD268 group (**e**). (**f**): Representative FCM images of H9C2 cells stained by DCFH-DA after being treated with various concentrations of FD274 and FD268. (**g**,**h**): The positive cell ratio (**g**) and mean fluorescence intensity (MFI) (**h**) of H9C2 cells from the FD274 group after staining with DCFH-DA, visualized by FCM (n = 3). (**i**,**j**): The positive cell ratio (**i**) and MFI (**j**) of H9C2 cells from the FD268 group after staining with DCFH-DA, visualized by FCM (n = 3). (**k**): Representative FCM images of H9C2 cell staining with Annexin V-FITC/PI after being treated with various concentrations of FD274 and FD268. (**l**): The expression levels of Bax, cleaved caspase-3, gp91phox, and Nrf2 in H9C2 cells after being treated with FD274 and FD268 were determined by Western blot. * *p* < 0.05, ** *p* < 0.01, *** *p* < 0.001, **** *p* < 0.0001.

The underlying mechanism of the different cardiotoxicity of FD274 and FD268 was further investigated in the H9C2 cell line. Firstly, the cell viability of H9C2 was assessed (Figure 2a,b). The cell viability of the FD274 group remained above 90% over the tested concentration range. For the FD268 group, the cell viability decreased to 88.43 ± 12.52% when treated with 0.5 μM FD268; after the treatment with 1 μM FD268, the cell viability significantly decreased to 51.62 ± 2.13% (*p* < 0.0001). DCFH-DA staining was then performed to visualize the ROS level in H9C2 cells. As shown by confocal laser scanning microscopy (CLSM) images (Figure 2c–e and Figure S4), no obvious ROS-positive cells were observed after being treated with FD274 over the tested concentration range, while a significantly high ROS-positive signal was observed after the treatment with 0.5 μM FD268, and the fluorescence intensity of the ROS was significantly higher than that in the control group (*p* < 0.01). Similarly, the flow cytometry (FCM) results further confirmed the low ROS-positive cell ratio and mean fluorescence intensity (MFI) of ROS in H9C2 cells after the treatment with FD274 (Figure 2f–h). By contrast, 0.2, 0.5, and 1 μM FD268 induced a significant increase in both the ROS-positive cell ratio and the MFI of ROS (Figure 2f,i,j). All these results confirmed the reduced toxic effect of FD274 in inducing ROS overproduction in H9C2 cells compared with that of FD268.

Apoptosis is one of the most common final steps that leads to cardiomyocyte death [26]. The apoptosis level of H9C2 cells was then quantified by Annexin V-FITC/PI staining after they were treated with various concentrations of FD274 and FD268. As shown in Figure 2k and Figure S5, FD274 did not affect the apoptosis level over the tested concentration range, while after the treatment with 1 μM FD268, H9C2 cells displayed significantly higher apoptosis levels compared with the control group (*p* < 0.001).

Nox2 (gp91phox), one of the NADPH oxidases, plays a predominant role in the accumulation of ROS in response to doxorubicin (DOX) exposure in mammalian hearts, which contributes to the generation of superoxide anion and hydrogen peroxide [7,10,27]. Nuclear factor erythroid 2-related factor 2 (Nrf2) is the major regulator of cellular responses against oxidative stress, and its down-regulation leads to a decrease in the activity of antioxidant enzymes [28,29]. After the treatment with 0.5 μM or 1 μM FD274 or FD268, the expression of gp91phox, Nrf2, and the apoptosis-related proteins in H9C2 cells was determined by Western blot analysis. As shown in Figure 2i, both FD274 and FD268 induced the up-regulation of NADPH oxidase gp91phox. FD274 did not influence the expression of apoptosis-related proteins Bax or cleaved caspase-3 or the antioxidant pathway protein Nrf2, while FD268 induced the up-regulation of Bax and cleaved caspase-3 and down-regulated the expression of Nrf2. These results suggest that the inhibition of Nrf2 and the activation of gp91phox resulted in increased oxidative stress as well as cell apoptosis in the FD268 group, while the preservation of the activity of Nrf2 mitigated oxidative stress and cell apoptosis in the FD274 group. All these results revealed the reduced cardiotoxicity of FD274 compared with FD268, as well as its underlying mechanism.

### 2.3. The Cardiotoxicity of FD274 in the HL-60 Xenograft Model After the Consecutive 20-Day Treatment Process

**Figure 3:**
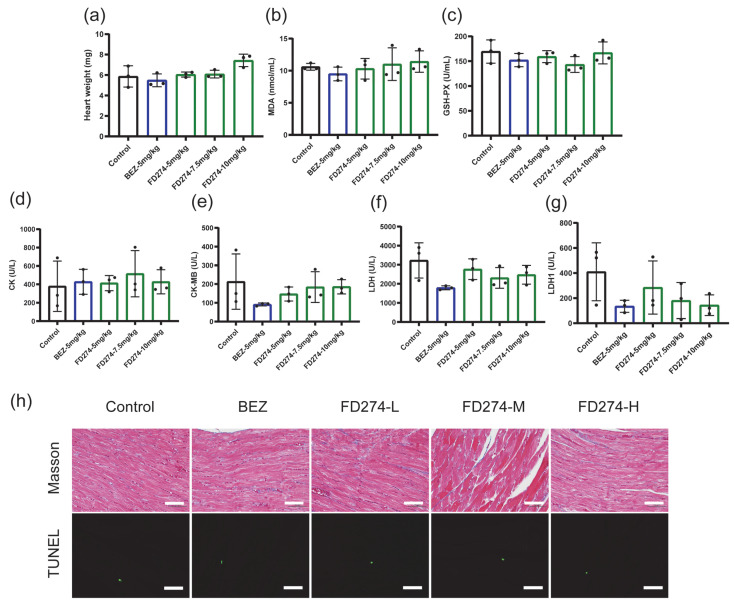
The cardiotoxicity of FD274 after the consecutive 20-day i.p. administration period in the HL-60 xenograft model (n = 3). (**a**): The heart weight of each group. (**b**–**g**): Levels of MDA (**b**), GSH-PX (**c**), CK (**d**), CK-MB (**e**), LDH (**f**), and LDH1 (**g**) in the mouse serum of each group. MDA: malondialdehyde; GSH-PX: glutathione peroxidase; CK: Creatine Kinase; CK-MB: Creatine Kinase, MB form; LDH: lactate dehydrogenase. (**h**): Masson’s trichrome and TUNEL staining of the heart sections of the HL-60 xenograft model from each group. Scale bar = 50 μm. BEZ: BEZ235 (5 mg/kg/day); FD274-L: FD274 (5 mg/kg/day); FD274-M: FD274 (7.5 mg/kg/day); FD274-H: FD274 (10 mg/kg/day).

We next investigated whether a long-term treatment process with FD274 would cause cardiotoxicity in tumor-bearing mice. BEZ235 served as the control for the treatment process as described in our previous work [25]. As shown in Figure 3, FD274 showed no adverse effects on heart weight, serum biochemical indexes including MDA and GSH-PX, or myocardial enzymes including Creatine Kinase (CK); Creatine Kinase, MB form (CK-MB); lactate dehydrogenase (LDH); and LDH1 over the tested dose range. Pathological examination revealed that no myocardial fibrosis or apoptosis occurred in any treatment group. All these results confirmed the negligible cardiotoxicity of FD274 after the 20-day treatment period.

### 2.4. The Synergistic Inhibitory Effects of FD274 with Gilteritinib in HL-60 and MV-4-11 Cell Lines

**Figure 4:**
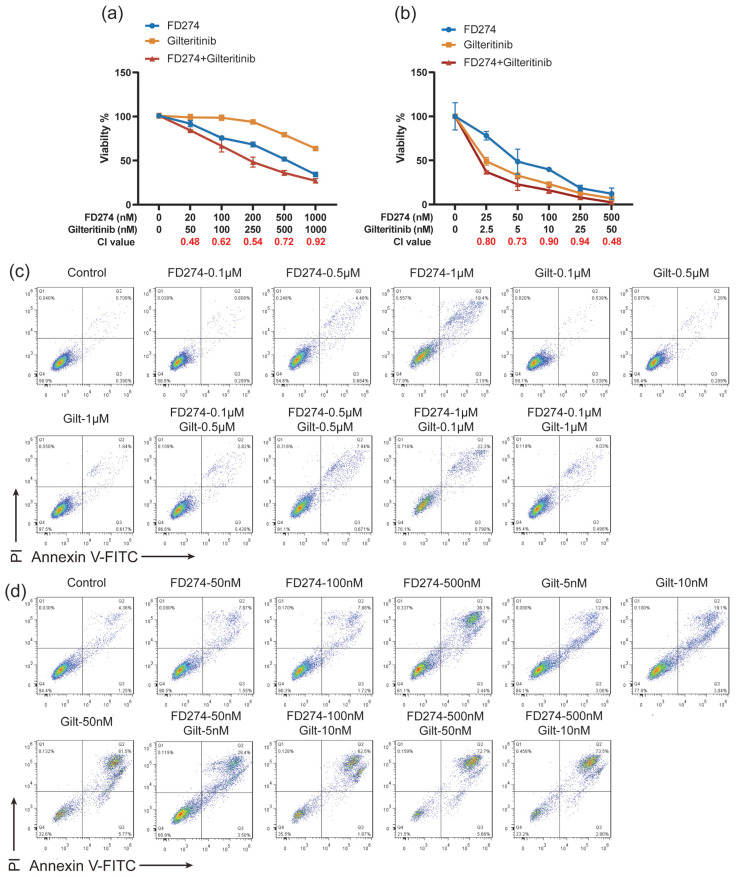
The synergistic cytotoxicity of FD274 with Gilteritinib in the HL-60 cell line and FLT3-ITD cell line MV-4-11 (n = 3). (**a**,**b**): Dose–response curves for HL-60 (**a**) and MV-4-11 (**b**) cells after treatment with various concentrations of FD274, Gilteritinib, or the combination of these two compounds. (**c**,**d**): Representative FCM images of HL-60 cells (**c**) and MV-4-11 cells (**d**) stained by Annexin V-FITC/PI after being treated with various concentrations of FD274, Gilteritinib, or the combination of these two compounds.

Encouraged by the above results, we explored the potential of FD274 for combination therapy with Gilteritinib. As shown in Figure 4a, both FD274 and Gilteritinib exhibited dose-dependent cytotoxic effects in HL-60 cells. When treated with 20~1000 nM FD274, the cell viability decreased from 91.977 ± 3.482% to 34.231 ± 2.599%; when treated with 50~1000 nM Gilteritinib, the cell viability decreased from 99.079 ± 3.494% to 63.721 ± 0.142%. The combination of FD274 and Gilteritinib caused higher inhibitory effects, with combination index (CI) values in the range of 0.48~0.92 (Figure 4a and Figure S6a), suggesting their synergistic effect. Similarly, the synergistic effects of FD274 with Gilteritinib were also observed in the FLT3-ITD cell line MV-4-11. As shown in Figure 4b, when treated with 25~500 nM FD274, the cell viability decreased from 78.090 ± 4.737% to 12.252 ± 6.464%; when treated with 2.5~50 nM Gilteritinib, the cell viability decreased from 49.061 ± 4.329% to 6.877 ± 1.081%; the combination of FD274 and Gilteritinib caused higher inhibitory effects, with CI values of 0.48~0.94 (Figure 4b and Figure S6b).

The synergistic effect of FD274 and Gilteritinib in inducing apoptosis was then investigated. As shown in Figure 4c and Figure S7a,c, 0.5 and 1 μM FD274 induced apoptosis of HL-60 cells, while Gilteritinib did not induce apoptosis over the tested concentration range. The apoptosis rates of the combination groups were 3.25 ± 0.25~22.57 ± 1.30%, with CI values of 0.50~0.98, which were significantly higher than those of the control group (1.32 ± 0.21%). The combination of 0.1~1 μM Gilteritinib with FD274 caused significantly higher apoptosis levels compared with the corresponding Gilteritinib-only group; the combination of 0.1~0.5 μM FD274 with 0.5~1 μM Gilteritinib caused significantly higher apoptosis levels compared with the corresponding FD274-only group. Similarly, the combination of FD274 with Gilteritinib enhanced the efficacy of inducing apoptosis in the MV-4-11 cell line compared with FD274 or Gilteritinib alone, with CI values in the range of 0.30~0.93 (Figure 4d and Figure S7b,c). These results suggest synergistic cytotoxic effects between FD274 and Gilteritinib.

To further investigate the underlying mechanism of the synergistic effect between FD274 and Gilteritinib, the expression of PI3K/AKT pathway proteins in MV-4-11 cells was assessed by Western blot analysis after treatment with FD274, Gilteritinib, or the combination of these two compounds. As shown in Figure S8, the Gilteritinib-only treatment up-regulated the phosphorylation level of AKT, which was down-regulated by the FD274-only treatment and the combination of FD274 with Gilteritinib. Additionally, the combination of these two compounds down-regulated the phosphorylation level of two downstream proteins of AKT, p70S6K, and 4EBP-1. These results suggest that the combination of FD274 with Gilteritinib reversed the activation of the PI3K/AKT pathway induced by Gilteritinib, meaning it can overcome Gilteritinib resistance.

## 3. Discussion

PI3K-targeted small molecules have been widely used for cancer therapy. Enlightened by the binding mode of GSK2126458 with PI3Kγ protein, our group developed a novel scaffold that conjugates a pyridinesulfonamide motif with 7-azaindole/azaindazole groups [21]. These compounds exhibited ideal binding modes and high enzymatic inhibitory effects against PI3Ks, with potent antiproliferative effects against a panel of human tumor cells, which contributes to the development of both pan-PI3K and isoform-specific PI3K inhibitors. Although FD223, the first star molecule of this class, has been reported as a promising PI3Kδ-selective inhibitor for the treatment of AML [30], its low inhibitory activity against the downstream targets of PI3K left room for further optimization. FD268, a representative of the pan-PI3K inhibitor candidate developed subsequently, was proven to inhibit the PI3K/AKT/mTOR signaling pathway and exhibited high antiproliferative effects against AML cells [31]. Shortly afterward, FD274, a bioisostere of FD268, was discovered as an equipotent dual inhibitor of PI3K and mTOR kinases with superior in vitro and in vivo antitumor activities [22]. Along with the prosperous development of lead compounds, the common occurrence of the cardiotoxicity of PI3K inhibitors in clinical trials raised concerns about medicinal chemistry strategies to attenuate toxicity as early as the development stage. From the viewpoint of the structure–cardiotoxicity relationship regarding informing the design strategy of novel PI3K inhibitors, we preliminarily reported the low, structure-dependent cardiotoxicity of FD274 among three bioisosteres under test by using zebrafish embryos [23]. Based on these results, the present study utilized mouse models to provide a comprehensive assessment of the cardiotoxicity of FD274 after absorption and metabolism. By using echocardiography, our results validated the low adverse effects of FD274 in inducing cardiac dysfunction. Furthermore, our results confirmed the negligible adverse effect of FD274 after the 20-day treatment process in HL-60 xenograft mice. All these results provide a more detailed cardiotoxic profile of FD274 for its potential clinical translation.

The PI3K/AKT pathway plays protective roles against oxidative stress and inflammatory injury [11,12,32,33], which promotes investigations into whether PI3K inhibitors would increase susceptibility to oxidative stress and subsequent cardiotoxicity. Based on our previous research, which found that 7-azaindazole-based PI3K inhibitors FD269 (the structure is shown in Figure S9) and FD268 up-regulate noxo1b and evoke oxidative stress injury [23], the present study further investigated the effects of 7-azaindazole-based PI3K inhibitors in inducing oxidative stress injury in a mouse model. Our results showed that the FD268 group exhibited a high level of MDA, the main product of lipid peroxidation [34], but low activity of glutathione oxidase, one of the endogenous antioxidants that can remove peroxides to prevent acute myocardial injury [7], which suggests the occurrence of oxidative stress. By contrast, both the MDA level and the glutathione oxidase level in the mouse serum of the FD274 group showed no difference compared with those of the control group. Nox2 is the major NADPH oxidase that transfers electrons from the cytosol to various intracellular and extracellular compartments [7,10]. Nuclear factor erythroid 2-related factor 2 (Nrf2) is the major regulator of cellular responses against oxidative stress, which can regulate the activity of antioxidant enzymes [28,29]. DOX can activate Nox2 and inhibit the Nrf2 antioxidant pathway, which leads to the generation of superoxide anion and hydrogen peroxide and the subsequent oxidative stress [27]. Our results showed that the oxidative stress injury induced by FD268 may be associated with the down-regulation of the Nrf2 antioxidant pathway and the up-regulation of Nox2 (gp91phox). Interestingly, FD274 also induced the up-regulation of Nox2 but, on the other hand, preserved the activity of Nrf2, which may explain the mitigation of oxidative stress and cardiac injury. All these results broaden our understanding of the mechanisms underlying the mitigation of oxidative stress and cell apoptosis induced by FD274. Future study is needed to investigate the mechanisms behind the oxidative stress related to the cardiotoxicity induced by 7-azaindazole-based PI3K inhibitors.

The ITD mutation of FLT3, observed in a subpopulation of AML patients, leads to its constitutive activation as well as the activation of its downstream signaling, such as PI3K/AKT, MAPK/ERK, and STAT5, which is associated with adverse prognosis [16,17]. Efforts to inhibit the PI3K/AKT/mTOR to address the resistance of FLT3 inhibitors demonstrated clinical benefits, which provides rationale for further trials on the combination of PI3K/AKT/mTOR inhibitors with FLT3 inhibitors [35]. In the present study, after comprehensively confirming the low cardiotoxicity of FD274, the efficacy of the combination of FD274 with FLT3 inhibitor Gilteritinib was investigated. Our results showed the synergistic inhibitory effects of FD274 with Gilteritinib on the HL-60 cell line and FLT3-ITD cell line MV-4-11. Additionally, our results revealed that the combination reversed the activation of AKT induced by Gilteritinib alone and down-regulated the downstream proteins of AKT in MV-4-11 cells. All these results provide preliminary evidence for the feasibility of combining FD274 with Gilteritinib to overcome drug resistance.

## 4. Materials and Methods

### 4.1. General

FD274 and FD268 were synthesized in our lab [21]. BEZ235 was purchased from MedChemExpress LLC (Shanghai, China), and Gilteritinib was purchased from Selleck Chemicals (Houston, TX, USA). Adult female C57BL/6 mice (6~8 weeks old) and male BALB/c nude mice (4~5 weeks old) were maintained in an animal room on a 12/12 h light/dark cycle with free access to water and food. All experiments were performed in strict accordance with the guidelines of the China Council on Animal Management. The protocols were approved by the Committee on the Ethics of Animal Experiments of Fudan University (Approval No. 202106046S). H9C2, HL-60, and MV-4-11 cell lines were purchased from Procell Life Science & Technology Co., Ltd. (Wuhan, China). Cells were cultivated in a 5% CO_2_ incubator at 37 °C with high-glucose DMEM (H9C2 cell line; Genombio, Hangzhou, China) or RPMI 1640 (HL-60 and MV-4-11 cell lines; Genombio, Hangzhou, China) containing 10% fetal bovine serum (Gibco, Grand Island, NY, USA) and 1× penicillin and streptomycin.

### 4.2. Drug Administration

FD274 and FD268 were dissolved in saline containing 40% PEG400 and 5% Tween-80 (*v*/*v*) to a dose of 7.5 mg/kg. C57BL/6 mice were randomly divided into three groups (n = 3)—control group, FD274 group, and FD268 group—and were respectively treated (i.p. administration) with the vehicle, FD274 solution, and FD268 solution.

The HL-60 xenograft model was established as described in the previous work [22]. Briefly, 5 × 10^6^ HL-60 cells were subcutaneously implanted in the right axilla region of BALB/c nude male mice. When the tumors reached 50~100 mm^3^, the mice were randomly divided into five groups (n = 3): the control group, FD274-5 mg/kg group, FD274-7.5 mg/kg group, FD274-10 mg/kg group, and BEZ235 group (n = 3). The control group was subjected to the i.p. administration of the vehicle, saline with 40% PEG400 + 5% Tween-80, every day. The FD274 groups were subjected to the i.p. administration of FD274 suspended in the vehicle at doses of 5 mg/kg/day, 7.5 mg/kg/day, or 10 mg/kg/day. The positive group was subjected to the i.p. administration of BEZ235 (MedChemExpress LLC, Shanghai, China) suspended in the vehicle at a dose of 5 mg/kg/day. After a consecutive 20-day administration, mice were euthanized, and the blood and the hearts of mice in each group were prepared for the serum biochemical analysis, histopathological analysis, and myocardial apoptosis determination.

### 4.3. Echocardiography

C57BL/6 mice were anesthetized by a small animal gas anesthesia machine, with 1~2% isoflurane in the room air. M-mode images were acquired by a Visual Sonic high-resolution 3100 system (FUJIFILM Visual Sonics, Toronto, ON, Canada). Ejection fraction (EF), fractional shortening (FS), left ventricular diameter (LVID), left ventricular end-systolic volume (LVESV), left ventricular end-diastolic volume (LVEDV), left ventricular anterior wall end-systolic dimension (LVAWs), LVAW-diastolic dimension (LVAWd), left ventricular posterior wall end-systolic dimension (LVPWs) and LVPW-diastolic dimension (LVPWd) were recorded.

### 4.4. Serum Biochemical Analysis

The blood of mice was collected and stood overnight at 4 °C and was centrifuged at 3000 rpm for 10 min. The determinations of the levels of glutathione peroxidase (GSH-PX, GPX) and malondialdehyde (MDA) in serum were performed by using the corresponding commercial kits according to the instructions. All kits were purchased from Jiancheng Institute of Biological Engineering (Nanjing, China).

### 4.5. Histopathological Analysis

The heart tissues were weighed by an analytical balance and were placed in 4% Paraformaldehyde Fix Solution (Sangon Biotech, Shanghai, China). The fixed heart was embedded in paraffin and was cut into 4 μm thick sections for H&E staining and Masson’s trichrome staining. Three 400× microscopic fields from each section were captured using a fluorescence microscope (Zeiss, Oberkochen, Germany). The H&E staining for the liver, spleen, lung, and kidney tissues was performed accordingly.

Myocardial apoptosis was examined by using a terminal deoxynucleotidyl transferase-mediated dUTP nick end labeling (TUNEL) method. A TUNEL Apoptosis Detection Kit (Servicebio, Wuhan, China) for detecting DNA fragmentation was used for tissue sections according to the manufacturer’s instructions. The fluorescent signal was captured using a fluorescence microscope.

### 4.6. Cell Viability Analysis

The cell viability was determined using a Cell Counting Kit-8 (CCK-8) kit (Vazyme, Nanjing, China). FD274, FD268, and Gilteritinib were first dissolved in DMSO (Sinopharm Chemical Reagent Co., Ltd., Beijing, China) to prepare 1000× stock solution and then were diluted with culture medium to prepare the corresponding working solutions. H9C2 cells were seeded with a density of 5000 cells/well in 96-well plates (Thermo Scientific Nunc, Waltham, MA, USA) and recovered overnight in the incubator. Then, cells were treated with 0.1, 0.2, 0.5, 1, and 2 μM FD274 and FD268 working solutions prepared as above, along with 0.1% DMSO as control. Blank wells added an equal amount of culture medium were set as background. After 24 h treatment, 10 μL CCK-8 solution was added to all treatment, control, and background wells, which were placed in the incubator for 2 h. The cell viability was calculated according to the absorbance read by a microplate reader (SYNERGY H1, BioTek, Winooski, VT, USA) at 450 nm of each well using the following formula: (OD_treatment_ − OD_background_)/(OD_control_ − OD_background_) × 100%.

The synergistic cytotoxicity of FD274 with Gilteritinib was investigated similarly. HL-60 cells were seeded with a density of 1 × 10^4^ cells/well in 96-well plates and stood overnight in the incubator. Then, cells were treated with FD274 (0, 20, 100, 200, 500, 1000 nM), Gilteritinib (0, 50, 100, 250, 500, 1000 nM), or the combination of the corresponding concentration gradients of these two compounds. MV-4-11 cells were seeded with a density of 1 × 10^4^ cells/well in 96-well plates and recovered overnight in the incubator. Then, cells were treated with 0, 25, 50, 100, 250, and 500 nM FD274; 0, 2.5, 5, 10, 25, and 50 nM Gilteritinib; or the combination of the corresponding concentration gradients of these two compounds. Blank wells added an equal amount of culture medium were set as background. After 48 h treatment, the CCK-8 assay was performed as described above.

### 4.7. Determination of Intracellular ROS Level

The FD274 and FD268 working solutions were prepared as described above. The intracellular ROS level was evaluated using DCFH-DA staining (Beyotime Biotechnology, Shanghai, China). H9C2 cells were seeded with a density of 1.5 × 10^5^ cells/plate in 35 mm Confocal Dishes (Biosharp, Hefei, China) and were treated with 0.1, 0.2, and 0.5 μM FD274 and FD268 working solutions. After 24 h treatment, DCFH-DA staining was performed according to the manufacturer’s instructions. The fluorescent signal was captured using confocal laser scanning microscopy (CLSM) (Nikon, Tokyo, Japan). The fluorescent intensity of each group was assessed using ImageJ 1.46r software (National Institutes of Health, Bethesda, MD, USA). Additionally, flow cytometry (FCM) was performed. H9C2 cells were seeded with a density of 6 × 10^5^ cells/plate in 60 mm dishes (Thermo Scientific Nunc, Waltham, MA, USA) and were treated with 0.1, 0.2, 0.5, and 1 μM FD274 and FD268 working solutions. After 24 h treatment, cells were collected, and DCFH-DA staining was performed according to the manufacturer’s instructions. The positive cell ratio and mean fluorescence intensity were detected by FCM (Beckman Coulter, Brea, CA, USA).

### 4.8. Determination of Apoptosis Level

The FD274, FD268, and Gilteritinib working solutions were prepared as described above. H9C2 cells were seeded with a density of 6 × 10^5^ cells/plate in 60 mm dishes and were treated with 0.1, 0.2, 0.5, and 1 μM FD274 and FD268 working solutions. After 24 h treatment, cells were collected, and Annexin V-FITC/PI staining was performed according to the manufacturer’s instructions of the kit (Beyotime Biotechnology, Shanghai, China). FCM was performed, and the apoptosis level in each group was measured as the ratio of the Annexin V-FITC-positive and both Annexin V-FITC- and PI-positive cells.

HL-60 cells were seeded with a density of 2 × 10^5^ cells/well in 6-well plates (Thermo Scientific Nunc, Waltham, MA, USA) and recovered overnight in the incubator. Then, cells were treated with FD274 (0, 100, 500, 1000 nM), Gilteritinib (0, 100, 500, 1000 nM), or the combination of certain concentration gradients of these two compounds. MV-4-11 cells were seeded with a density of 2 × 10^5^ cells/well in 6-well plates and stood overnight in the incubator. Then, cells were treated with FD274 (0, 50, 100, 500 nM), Gilteritinib (0, 5, 10, 50 nM), or the combination of certain concentration gradients of these two compounds. After 48 h treatment, cells were collected, and Annexin V-FITC/PI-staining FCM analysis was performed as described above.

### 4.9. Western Blot

The FD274, FD268, and Gilteritinib working solutions were prepared as described above. H9C2 cells were seeded with a density of 6 × 10^5^ cells/plate in 60 mm dishes and were treated with 0.5 and 1 μM FD274 and FD268 working solutions. After 24 h treatment, cells were collected and lysed using cell lysis buffer (CST; Shanghai, China) containing 1× phosphatase and protease inhibitor Cocktail (Roche; Basel, Switzerland) to obtain proteins. MV-4-11 cells were seeded with a density of 2 × 10^5^ cells/well in 6-well plates and recovered overnight in the incubator. Then, cells were treated with FD274 (0, 50, 100, 500 nM), Gilteritinib (0, 5, 10, 50 nM), or the combination of certain concentration gradients of these two compounds. After 48 h treatment, cells were collected, and proteins were then obtained as described above.

Protein concentration was determined using the BCA protein assay kit (Thermo Scientific, Waltham, MA, USA). The protein samples were subjected to SDS-PAGE gel with a sample volume of 30 μg. After the SDS-PAGE was finished, proteins were transferred to PVDF membranes (Sigma-Aldrich, Steinheim, Germany). The membranes were blocked with 5% BSA in TBST for 1.5 h and were then incubated with primary antibody for 14~16 h at 4 °C. After washing 3 times with TBST, the membrane was treated with secondary antibody (1:3000 dilution, CST, Kansas City, MO, USA) at room temperature for 1 h. The target bands were visualized using an enhanced ECL detection kit with ChemiDocTM MP imaging system (BioRad, Shanghai, China).

### 4.10. Statistical Analysis

The synergistic effects of FD274 with Gilteritinib were evaluated by combination index (CI). CI was calculated by CompuSyn 1.0 software (ComboSyn Inc., Paramus, NJ, USA). A CI less than 1 indicates synergy effects, and a CI greater than 1 indicates antagonistic effects.

Data were expressed as mean ± standard deviation (SD). Statistical analysis was performed using Graphpad Prism 8.3.0 (GraphPad Software, Inc., La Jolla, CA, USA). Statistical significance among three or more groups was determined by one-way ANOVA, and Dunnett was performed to correct for multiple comparisons (* *p* < 0.05, ** *p* < 0.01, *** *p* < 0.001, **** *p* < 0.0001).

## 5. Conclusions

The assessment of the cardiotoxicity of PI3K inhibitors in the drug development stage is of great importance for their potential clinical translation. Our results confirmed the low adverse effects of FD274 associated with cardiac dysfunction, oxidative stress, and myocardial injury in the C57BL/6 mouse model and in the H9C2 cell line. The mitigation of oxidative stress and cell apoptosis induced by FD274 may be ascribed to the preservation of the activity of Nrf2. The negligible adverse effect of FD274 was further validated in HL-60 xenograft mice after a 20-day treatment process. Additionally, we showed the potential of the combined therapy of FD274 with Gilteritinib for AML. These results revealed that the candidate FD274, with high antitumor efficacy, demonstrated low cardiotoxicity and is a great prospect for combined therapy that warrants further development.

## Data Availability

Data are available within the article or its Supplementary Materials.

## Supplementary Materials

The following supporting information can be downloaded at: https://www.mdpi.com/article/10.3390/molecules30112347/s1, Table S1: Pharmacokinetic profile of FD274 in rats. Figure S1: Cardiac function parameters of mice on the fourth day after being treated with vehicle (control), FD274, and FD268 (n = 3). LVAWs: left ventricular anterior wall end-systolic dimension; LVAWd: LVAW-diastolic dimension; LVPWs: left ventricular posterior wall end-systolic dimension; LVPWd: LVPW-diastolic dimension. Figure S2: TUNEL staining of the heart sections of mice on the fourth day after being treated with vehicle (control), FD274, and FD268. Scale bar = 50 μm. Figure S3: H&E staining of the liver, spleen, lung, and kidney sections of mice on the fourth day after being treated with vehicle (control), FD274, and FD268. Scale bar = 50 μm. Figure S4: Representative CLSM images of H9C2 cells stained by DCFH-DA after being treated with various concentrations of FD274 (a) and FD268 (b), scale bar = 100 μm. Figure S5: The apoptosis level of FD274 group (a) and FD268 group (b) was measured as the ratio of Annexin V-positive cells and both Annexin V- and PI-positive cells (n = 3). *** *p* < 0.001. Figure S6: The normalized isobologram for the combination of FD274 and Gilteritinib against HL-60 (a) and MV-4-11 (b) generated by CompuSyn software. Figure S7: The apoptosis levels of HL-60 (a) and MV-4-11 (b) were measured as the ratio of Annexin V-positive cells (early apoptosis) and both Annexin V- and PI-positive cells (late apoptosis) (n = 3). (c,d): The normalized isobologram for the combination of FD274 and Gilteritinib against HL-60 (c) and MV-4-11 (d) generated by CompuSyn software. ** *p* < 0.01 vs. control group, **** *p* < 0.0001 vs. control group; ### *p* < 0.001 vs. FD274 alone, #### *p* < 0.0001 vs. FD274 alone; $ *p* < 0.05 vs. Gilteritinib alone, $$$ *p* < 0.001 vs. Gilteritinib alone, $$$$ *p* < 0.0001 vs. Gilteritinib alone. Figure S8: The expression levels of AKT, p-AKT, p-mTOR, p70S6K, p-p70S6K, 4E-BP1, and p-4E-BP1 in MV-4-11 cells after being treated with FD274, Gilteritinib, and the combination of these two compounds were determined by Western blot. Figure S9. The chemical structure of FD269.

## Abbreviations

The following abbreviations are used in this manuscript:

AML	acute myelogenous leukemia
PI3K	Phosphoinositide-3-kinases
ROS	reactive oxygen species
NADPH	Nicotinamide Adenine Dinucleotide Phosphate Hydrogen
FLT3	Fms-like tyrosine kinase 3
ITD	internal tandem duplication
MDA	malondialdehyde
GSH-PX	glutathione peroxidase
LVIDs	left ventricular internal dimension systole
LVIDd	left ventricular internal dimension diastole
LVESV	left ventricular end-systolic volume
LVEDV	left ventricular end-diastolic volume
EF	ejection fraction
FS	fractional shortening
TUNEL	terminal deoxynucleotidyl transferase-mediated dUTP nick end labeling
MFI	mean fluorescence intensity
DCFH-DA	2′,7′-Dichlorofluorescein diacetate
FCM	flow cytometry
CLSM	confocal laser scanning microscopy
H&E	hematoxylin–eosin
CK	Creatine Kinase
CK-MB	Creatine Kinase, MB form
LDH	lactate dehydrogenase
Nrf2	Nuclear factor erythroid 2-related factor 2
CI	combination index
