# Supplementary material for: A PI3K Inhibitor with Low Cardiotoxicity and Its Synergistic Inhibitory Effect with Gilteritinib in Acute Myelogenous Leukemia (AML) Cells
Source: Molecules. 2025 May 27;30(11):2347. doi: 10.3390/molecules30112347 (PMC12156206; doi:10.3390/molecules30112347)
Supplement: Supplementary file 1 [file molecules-30-02347-s001.zip › molecules-3613415-supplementary.pdf]

A PI3K Inhibitor with Low Cardiotoxicity and Its Synergistic Inhibitory Effect with Gilteritinib in Acute Myelogenous Leukemia (AML) Cells

Tianze Wu [1], Yi Chen [1], Yimin Gong [1], Mingzhu Lu [1], Chengbin Yang [1], Yannan Yang [2], Yun Ling [1] and Yaming Zhou [1,*]

[1] Department of Chemistry, Fudan University, Shanghai 200433, China; 19110220003@fudan.edu.cn (T.W.); 18110220046@fudan.edu.cn (Y.C.); 22110220126@m.fudan.edu.cn (Y.G.); 20110220017@fudan.edu.cn (M.L.); ycb@fudan.edu.cn (C.Y.); yunling@fudan.edu.cn (Y.L.)

[2] South Australian immunoGENomics Cancer Institute, The University of Adelaide, Adelaide, SA 5005, Australia; yannan.yang@adelaide.edu.au

* Correspondence: ymzhou@fudan.edu.cn

Table S1. Pharmacokinetic profile of FD274 in rats.[1]

iv (2 mg/kg)[a]		po (10 mg/kg)[b]	
$T_{1/2}$ (h)	2.29	$T_{1/2}$ (h)	4.48
V_z (mL/kg)	7260	T_{max} (h)	2.80
CL (mL h^{-1} kg^{-1})	2220	C_{max} (ng/mL)	15.5
AUC_{0-t} (h ng/mL)	898	AUC_{0-t} (h ng/mL)	104
$AUC_{0-\infty}$ (h ng/mL)	903	$AUC_{0-\infty}$(h ng/mL)	121
MRT_{INF} (h)	0.593	F (%)	2.68

[a] iv formulation: 10% DMSO in Water + 90% saline.

[b] po formulation: 0.1% PEG400 + 0.5% CMC-Na in Water. Three SD male rats for iv and 5 SD male rats for po

Figure S1 Cardiac function parameters of mice on the fourth day after respectively treated with vehicle (control), FD274, and FD268 (n = 3). LVAWs: left ventricular anterior wall end-systolic dimension; LVAWd: LVAW-diastolic dimension; LVPWs: left ventricular posterior wall end-systolic dimension; LVPWd: LVPW-diastolic dimension.

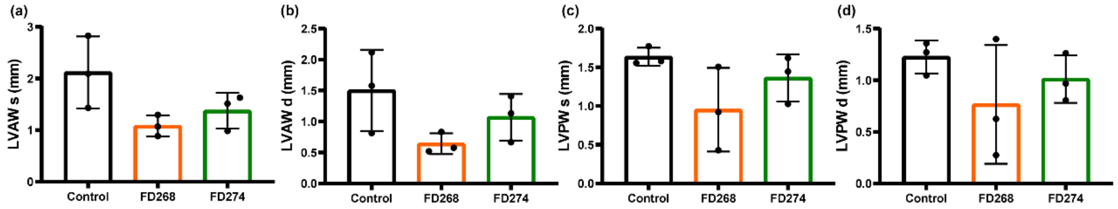

Figure S2 TUNEL staining of the heart sections of mice on the fourth day after respectively treated with vehicle (control), FD274, and FD268. Scale bar = 50 μm

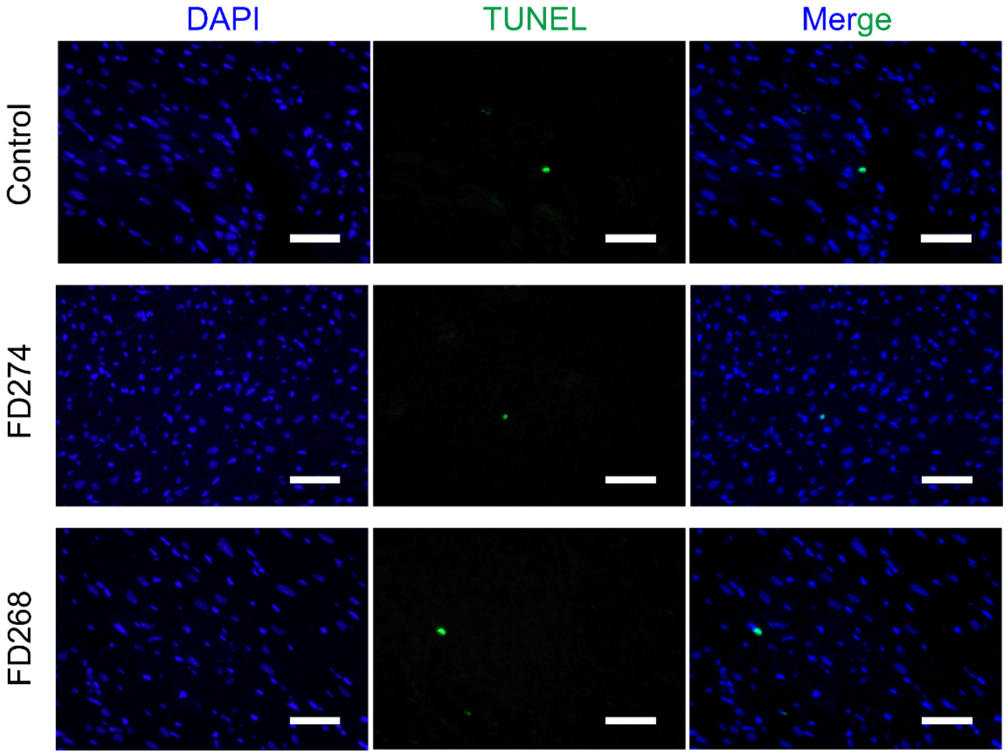

Figure S3 H&E staining of the liver, spleen, lung, and kidney sections of mice on the fourth day after respectively treated with vehicle (control), FD274, and FD268. Scale bar = 50 μm.

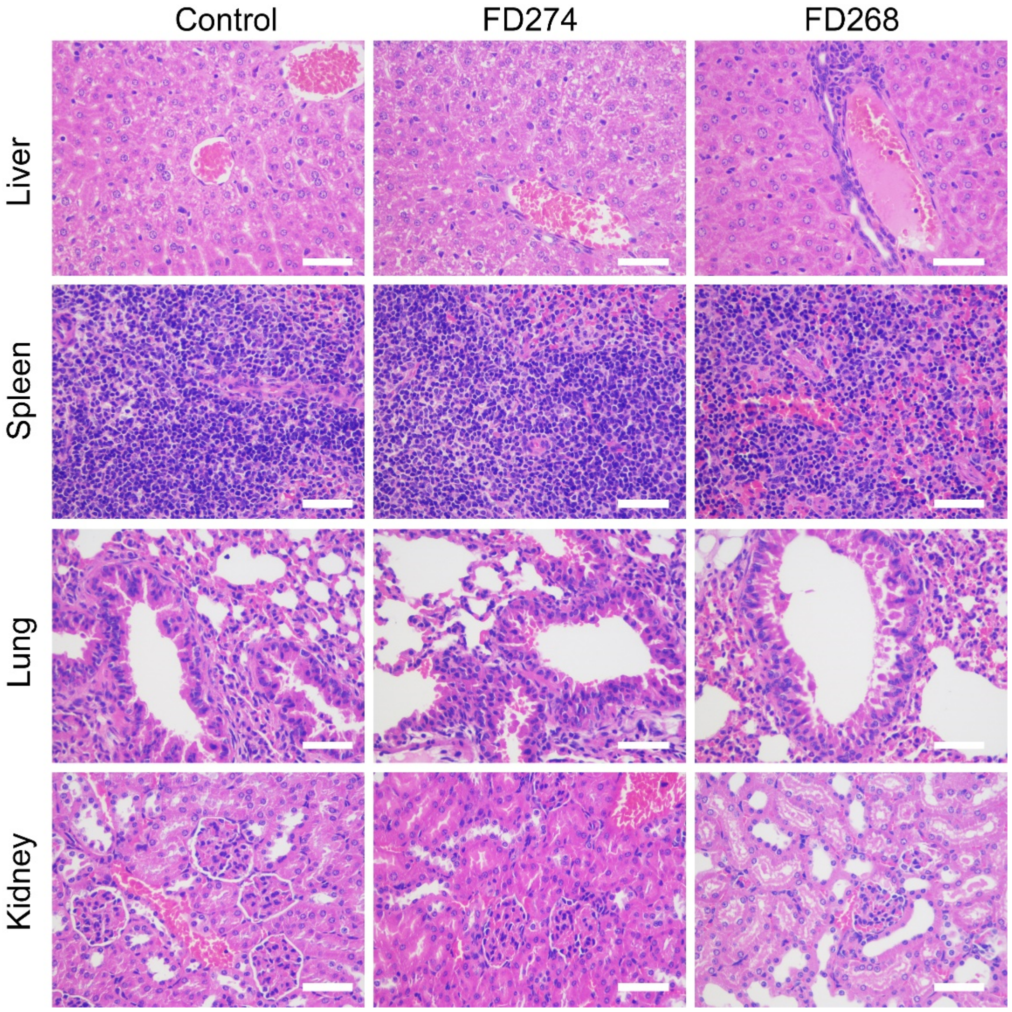

Figure S4 Representative CLSM images of H9C2 cells stained by DCFH-DA after respectively treated with various concentrations of FD274 (**a**) and FD268 (**b**), scale bar = 100 μm.

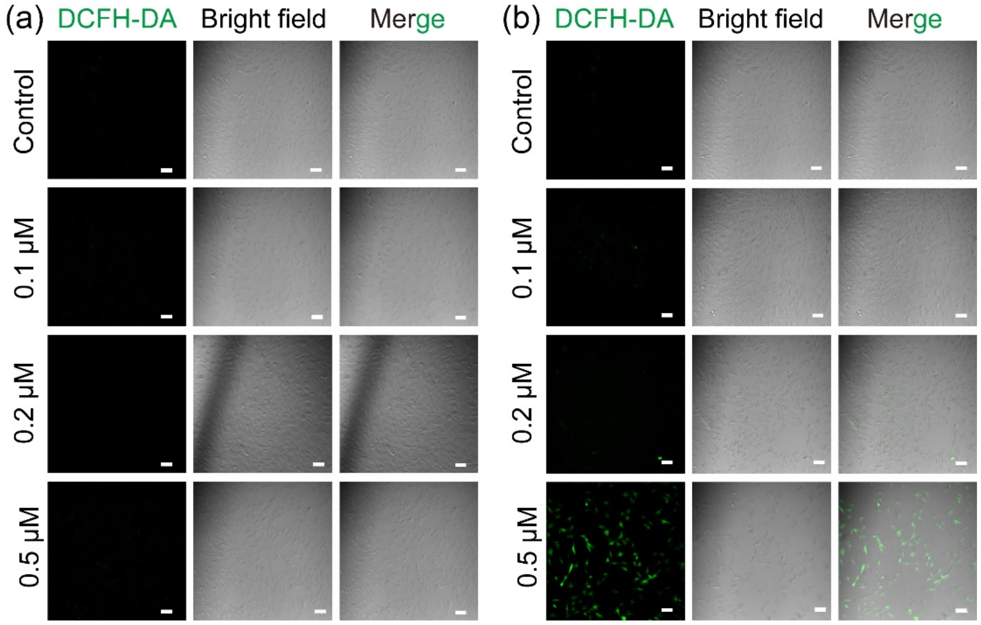

Figure S5 The apoptosis level of FD274 group (**a**) and FD268 group (**b**) was measured as the ratio of Annexin V-positive cells and both Annexin V- and PI-positive cells (n = 3). ***p < 0.001

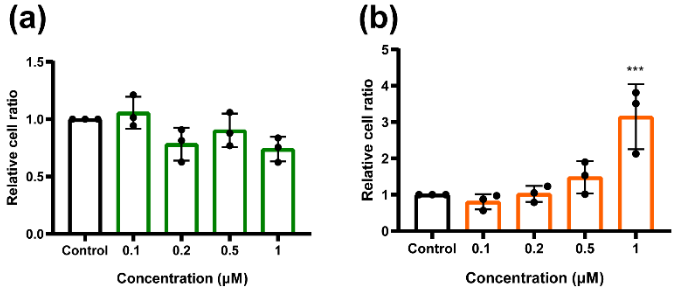

Figure S6 The normalized isobologram for the combination of FD274 and Gilteritinib against HL-60 (**a**) and MV-4-11 (**b**) generated by CompuSyn software.

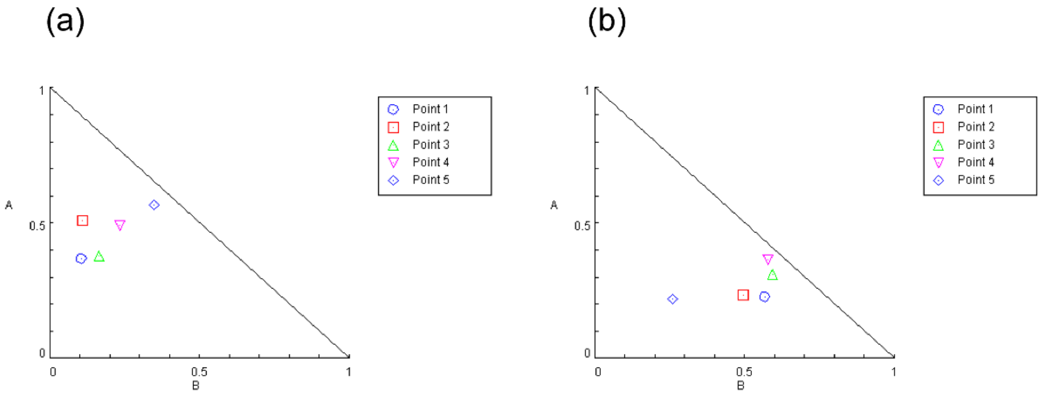

Figure S7 The apoptosis level of HL-60 (**a**) and MV-4-11 (**b**) was measured as the ratio of Annexin V-positive cells (early apoptosis) and both Annexin V- and PI-positive cells (late apoptosis) (n = 3).**c**-**d**: The normalized isobologram for the combination of FD274 and Gilteritinib against HL-60 (**c**) and MV-4-11 (**d**) generated by CompuSyn software. ***p* < 0.01 vs control group, *****p* < 0.0001 vs control group; ### *p* < 0.001 vs FD274 alone, #### *p* < 0.0001 vs FD274 alone; $ *p* < 0.05 vs Gilteritinib alone, $$$ *p* < 0.001 vs Gilteritinib alone, $$$$ *p* < 0.0001 vs Gilteritinib alone

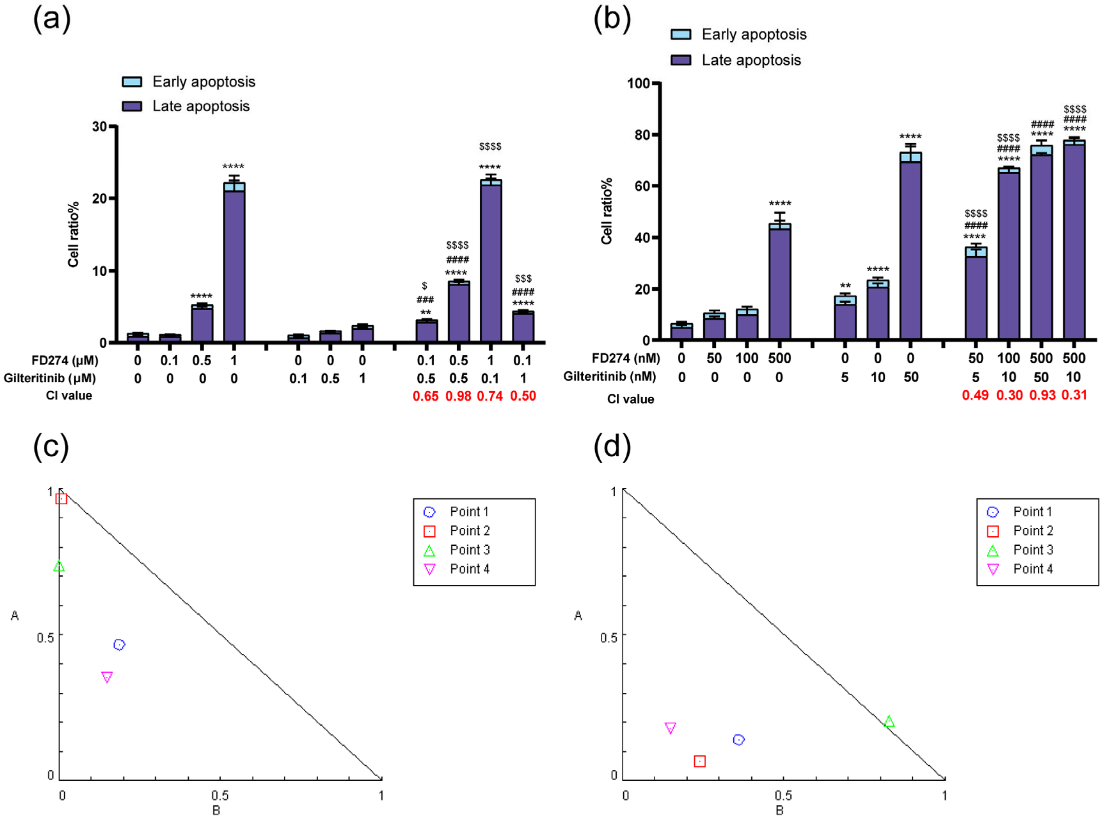

Figure S8 The expression levels of AKT, p-AKT, p-mTOR, p70S6K, p- p70S6K, 4E-BP1, and p-4E-BP1in MV-4-11 cells after respectively treated with FD274, Gilteritinib, and the combination of these two compounds were determined by western blot.

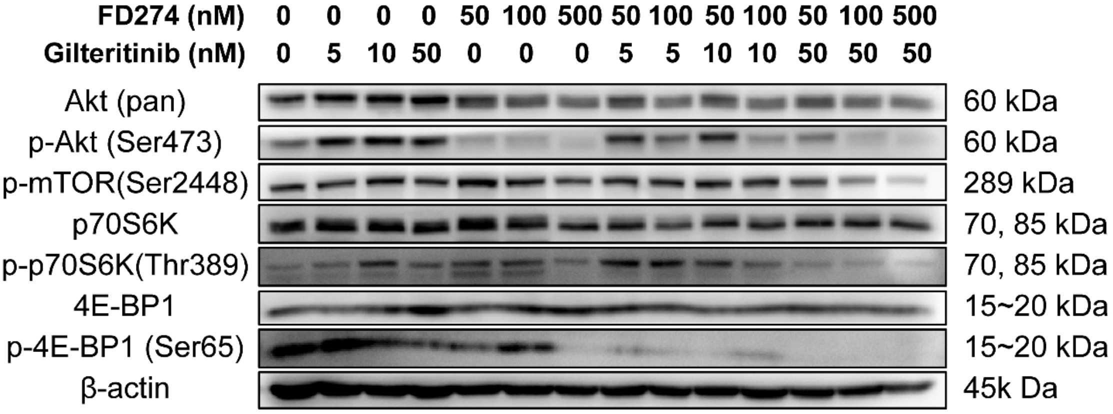

Figure S9. The chemical structure of FD269.

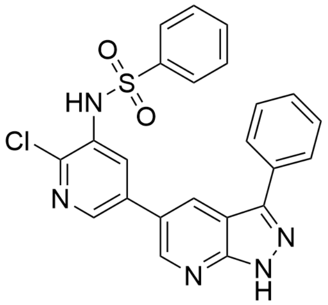

FD269

References

[1] Yang, C.; Chen, Y.; Wu, T.; Gao, Y.; Liu, X.; Yang, Y.; Ling, Y.; Jia, Y.; Deng, M.; Wang, J.; Zhou, Y., Discovery of N-(2-chloro-5-(3-(pyridin-4-yl)-1H-pyrazolo[3,4-b]pyridin-5-yl)pyridin-3-yl)-4-fluorobenzenesulfonamide (FD274) as a highly potent PI3K/mTOR dual inhibitor for the treatment of acute myeloid leukemia. *Eur. J. Med. Chem.* **2023,** *258,* 115543.
